# A tool for protected area management: multivariate control charts ‘cope’ with rare variable communities

**DOI:** 10.1002/ece3.585

**Published:** 2013-05-01

**Authors:** Thomas B Stringell, Roger N Bamber, Mark Burton, Charles Lindenbaum, Lucie R Skates, William G Sanderson

**Affiliations:** 1 Marine and Freshwater Science Group, Natural Resources WalesMaes y Ffynnon, Ffordd Penrhos, Bangor, LL57 2DN, U.K; 2 Centre for Ecology & Conservation, University ExeterCampus Penryn, Cornwall, TR10 9EZ, U.K; 3 ARTOO, Ocean Quay MarinaBelvidere Road, Southampton, SO14 5QY, U.K; 4 Skomer Marine Nature Reserve, Natural Resources WalesFisherman's Cottage, Marloes, Haverfordwest, SA62 3BJ, U.K; 5 Marine Monitoring Service, Environment AgencyKingfisher House, Orton Goldhay, Peterborough, PE2 5ZR, U.K; 6 Centre for Marine Biodiversity and Biotechnology, John Muir BuildingHeriot Watt University, Edinburgh, EH14 4AS, U.K

**Keywords:** Conservation management, Habitats Directive, indicator, lagoon, sustainable use, Water Framework Directive

## Abstract

Performance assessment, impact detection, and the assessment of regulatory compliance are common scientific problems for the management of protected areas. Some habitats in protected areas, however, are rare and/or variable and are not often selected for study by ecologists because they preclude comparison with controls and high community variability makes meaningful change detection difficult. Shallow coastal saline lagoons are habitats that experience comparatively high levels of stress due to high physical variability. Lagoons are rare, declining habitats found in coastal regions throughout Europe (and elsewhere) where they are identified as one of the habitats most in need of protected area management. The infauna in the sediments of 25 lagoons were sampled. Temporal and spatial variation in three of these [protected] lagoons was investigated further over 5 years. In a multivariate analysis of community structure similarities were found between some lagoons, but in other cases communities were unique or specific to only two sites. The protected lagoons with these unique/specific communities showed significant temporal and spatial variation, yet none of the changes observed were attributed to human impacts and were interpreted as inherent variability. Multivariate control charts can operate without experimental controls and were used to assess community changes within the context of ‘normal’ lagoon variability. The aim of control chart analysis is to characterize background variability in a parameter and identify when a new observation deviates more than expected. In only 1 year was variability more than expected and corresponded with the coldest December in over 100 years. Multivariate control charts are likely to have wide application in the management of protected areas and other natural systems where variability and/or rarity preclude conventional analytical and experimental approaches but where assessments of condition, impact or regulatory compliance are nonetheless required.

## Introduction

As a system of management, protected areas on land and/or sea are used to safeguard and maintain biological diversity and natural and associated cultural resources (cf. Pomeroy et al. 2004). The science of understanding the effectiveness of these protected areas, impacts upon them and regulatory compliance within them is widely recognized as providing a crucial role in the achievement of their objectives (e.g., Pomeroy et al. 2004; Sobel and Dalgren 2004; Gaston et al. 2008). In this context, studies have often sought to establish whether predefined standards have been achieved, whether indicative of impact, environmental quality or other set standards (e.g., Hilborn and Walters 1981; Hellawell 1991; Nijboer et al. 2004; Ruellet and Dauvin 2007). Field experimental approaches have also been used in which replicated reserve effect treatments are compared to controls (e.g., Eberhardt and Thomas 1991; Lester et al. 2009) in randomized designs that approximate to Before-After-Control-Impact (BACI) experiments (Underwood 1990, 1997). Overall, critics of monitoring programs consider that too many are “passive, mindless and lacking questions” (Lindenmayer and Likens 2010) and arguably, therefore, BACI-like designs that test hypotheses are regarded as ‘best practice’, although they may be hard to apply to rare habitats if suitable controls cannot be found.

Coastal saline lagoons are rare, threatened habitats in Europe (Council of the European Communities 1992) and a number of protected species are mainly or entirely restricted to them (Bamber et al. 1992; Gilliland and Sanderson 2000). Consequently, these habitats are identified as priorities for conservation within protected areas (Council of the European Communities 1992), necessitating applied science to assess their status and the effectiveness of management measures taken to protect them (e.g., Council of the European Communities 1992: Articles 6 and 17 of Council Directive 92/43EEC; European Commission 2000: Council Directive 2000/60/EC).

Lagoons, however, challenge those seeking to establish monitoring programs because they are physically highly variable, making it hard to set reference values or targets and to detect changes above background variation or ‘noise’ (Pérez-Ruzafa et al. 2007). This is especially true when using a low sampling frequency, which is typical of financially constrained government monitoring programs (Lucas et al. 2006). Variability in salinity, as well as in temperature, dissolved oxygen and pH, imparts comparatively severe environmental stresses (Lucas et al. 2006; Pérez-Ruzafa et al. 2007; Barnes et al. 2008) that are thought to be responsible for the development of specialist communities in lagoons (Bamber et al. 1992). Lagoonal assemblages also show high natural variability in response to environmental variability (Rosa and Bemvenuti 2006; Pérez-Ruzafa et al. 2007; Como and Magni 2009), to an extent that would be judged as negative if caused by anthropogenic activities (Breber et al. 2007). Logic dictates that a robust sampling regime is required that will provide enough statistical power to detect significant changes above this natural ‘noise’ (Pérez-Ruzafa et al. 2007), but this requirement must be balanced with the need to minimize damage to what are often small (<30 ha) habitats.

Control charts, a univariate analytical method that has its roots in industrial quality control (Allen et al. 1997), have recently been adapted for ecological applications within a multivariate framework and are particularly well suited to species abundance data that require no specific distributions and can be used with any distance measure (Anderson and Thompson 2004). The aim of multivariate control chart (MCC) analysis is to characterize background variability in the parameter(s) of interest and to identify when a new observation deviates more than expected from background; this is accomplished by reference to ‘control limits’ that represent the normal variation of the parameter so that an observation falling outside of these limits can be said to be ‘out of control’ (Trexler and Goss 2009).

The aim of this study was to explore how traditional and MCC approaches to the assessment of management success would apply to rare, variable habitats in protected areas. In this study similarities in infaunal community structure between saline lagoons were explored. The infaunal communities within saline lagoons were then examined using conventional multivariate methods against the null hypothesis of no significant inherent spatial or temporal variability in the infaunal community. We also tested to ensure that any significant variability was attributable to differences in species abundances and not within-group multivariate dispersion. MCCs were then applied to the same data to test the second null-hypothesis that there would be no differences in the conclusions reached about significant temporal variability. In this article, we offer a tool to environmental managers who are beset by significant variation in protected communities, with little scope for comparison elsewhere, but are nevertheless compelled by logic and legislation to make assessments.

## Material and Methods

### Study sites

**Figure 1:**
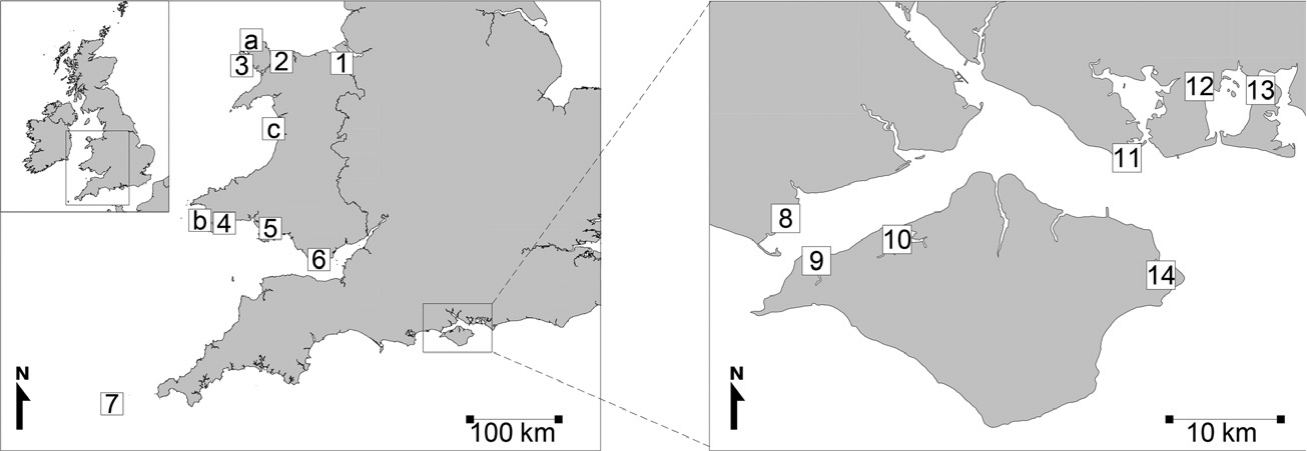
Study sites: a. Cemlyn, b. Pickleridge, c. Morfa Gwyllt, 1. Connah's Quay, 2. Morfa Aber East & West, 3. Inland Sea, 4. Carew Castle. 5. Penclacwydd, 6. Aberthaw, 7. Bryher, 8. Keyhaven, Oxey South, Eight-Acre Pond, Normandy Farm, Pennington, 9. Yar Lagoon, 10. Newtown, 11. Gilkicker, 12. Shut Lake, 13. Langstone Oyster Bed, 14. Bembridge Harbour Lagoon, East Harbour Lagoon, Harbour Farm Lagoons 1 and 2.

**Figure 2:**
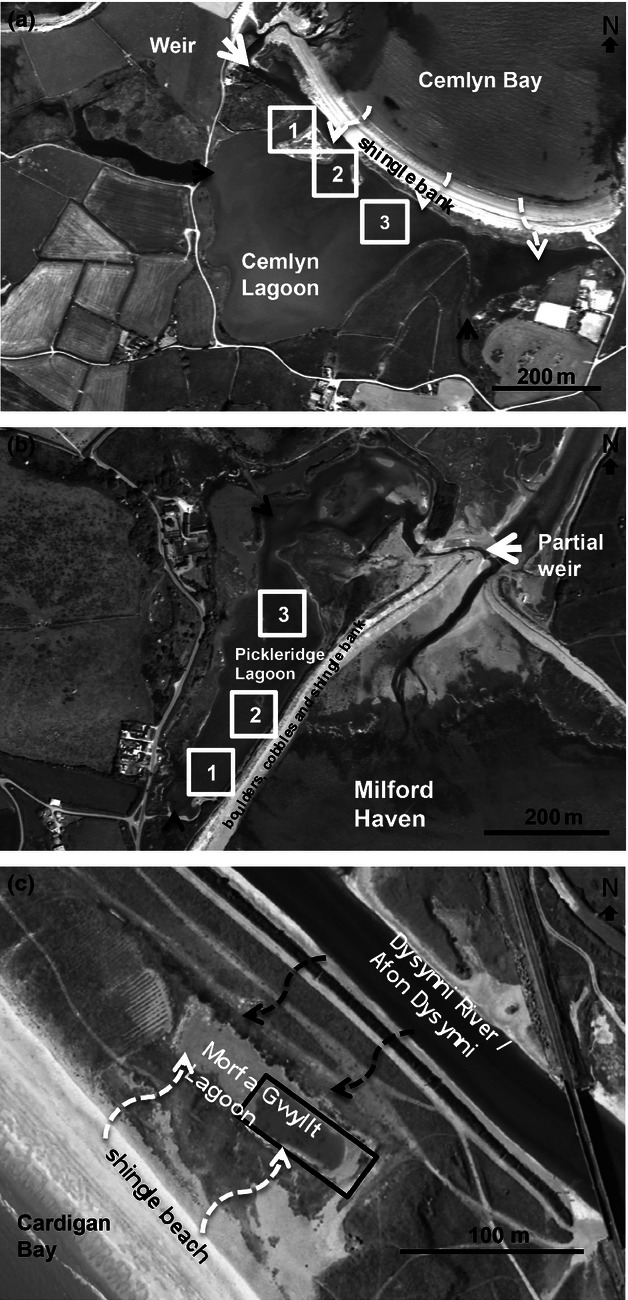
Orthorectified aerial photographs of: (a) Cemlyn lagoon with sampling plots (1–3) overlayed, (b) Pickleridge lagoon with sampling plots (1–3) overlayed, (c) Morfa Gwyllt lagoon with single sampling plot indicated by box (image from a dry period when water levels were low). Seawater input and freshwater input indicated by light and dark arrows, respectively. Wavy dotted arrows indicate possible percolation. Aerial photography in 2006 at 40 cm resolution (© COWI A/S, licensed by Welsh Government DEPC).

Twenty-five lagoon and lagoon-like habitats were sampled between 1998 and 2006 (Fig. 1). Of these, Cemlyn, Pickleridge, and Morfa Gwyllt lagoons (53.409°N, 004.513°W; 51.718°N, 005.171°W and 52.605°N, 004.121°W, respectively; Figs. 1, 2) were repeatedly sampled between 2006 and 2010 (below). Seawater and freshwater inputs to these sites were via weirs, streams, and drainage ditches illustrated in Figure 2 and also from rainfall and percolation through shingle. Maximum water depths varied between 0.7 and 1.5 m in these low volume lagoonal basins and the predominant sediments were typically mud or muddy sand overlying or among shingle (Bamber et al. 2001).

### Collation of infaunal community data

Infaunal data were collated from surveys undertaken by the authors in 25 lagoons between 1998 and 2006. Ten 0.005 m^2^ cores were collected from each lagoon, sieved through a 0.5 mm mesh, and then pooled. A small boat was used at Cemlyn and Pickleridge therefore sampling procedures were undertaken using a 0.025 m^2^ Ekman grab and two samples pooled to produce the same total sample area of 0.05 m^2^. In December 2006, Cemlyn, Pickleridge, and Morfa Gwyllt were resampled using the same methods. All sampling had typically been between September and December. Cemlyn was also sampled in March and August 2006 to introduce some seasonality into the lagoon comparisons.

### Repeat sampling

Three sampling plots in Cemlyn and Pickleridge (Fig. 2a and b) were chosen to capture the range of potential benthic variation that might occur due to point sources of seawater and freshwater input and to minimize the risk of widespread disturbance. At the smaller Morfa Gwyllt no potential input gradients were likely and a single sample plot was therefore selected (Fig. 2c). Sampling plots were selected in the deepest, more stable parts of the lagoons to avoid the most extreme short-term variation that might occur in the shallowest, occasionally dry, parts of the lagoons (see Fig. 2c for Morfa Gwyllt during a dry period).

Benthic samples were taken in December 2006 to 2010. The same sampling plots (Fig. 2) at Cemlyn, Pickleridge and Morfa Gwyllt had previously been sampled in December 1998 (see above) and the same sampling procedures were followed except that four replicate infaunal samples were collected at each Cemlyn and Pickleridge sample plot during the repeat sampling. All samples were preserved in 4% formalin solution, then transferred to a laboratory where biota were sorted and enumerated to the lowest possible taxonomic level (see CSEMP 2012).

### Data treatment and statistical analysis

Limited aggregations or exclusions were made in the data to avoid taxonomic inconsistencies and avoid the inclusion of species that could not be used quantitatively (Nematodes, Ostracods) or those whose populations were not directly responsive to the environmental conditions of the lagoon because key life history stages occurred elsewhere (Chironomids). Throughout the present study multivariate analyses were conducted on Bray-Curtis similarity coefficients of species abundance data using PRIMER v6 and PERMANOVA+ software (Clarke and Gorley 2006; Anderson et al. 2008). All species abundance data were fourth root-transformed so that the analyses represented the response of the whole community better and limited the influence of species exhibiting very high numerical dominance (up to three order of magnitude difference).

Hierarchical clustering (by group-average-fusion) of collated lagoon data was used to examine multivariate structure and a Similarity Profile (SIMPROF) permutation procedure was used to test the significance (at the 5% level) of the clusters.

Variation between sample plots and years (2006–2010) were tested at Cemlyn and Pickleridge as random and fixed effects (respectively) in a 2-way crossed design using Permutational Multivariate Analysis of Variance (PERMANOVA) with pair-wise comparisons between years. The variation between years at Morfa Gwyllt was tested as a fixed effect in a one-way design and pair-wise comparisons were also made between years. Tests were based on 9999 permutations of residuals under a reduced model for Cemlyn and Pickleridge and based on unrestricted permutations of raw data for Morfa Gwyllt. Type III Sums of Squares were used for each protected lagoon analysis. Permutational tests of homogeneity of multivariate dispersions (PERMDISP) were undertaken for each main effect (sample plot and years) to elucidate whether any significant variation from the PERMANOVAs was attributable to differences in dispersion of samples.

### Multivariate control charts

Multivariate control charts (MCC) were constructed (ControlChart.exe; Anderson 2005, 2008) for each protected lagoon and its sample plots. At Morfa Gwyllt data were not collected in distinct plots so replicates collected at the same time throughout the site were randomly assigned to three pseudo-sample plots to enable a similar control chart to be constructed.

To explore deviation from expectation, two MCC measures were applied:

1. deviation of a community at a sample plot at time *t* from a centroid obtained using a baseline derived from the first two observations from that lagoon;
2. the deviation of a community at a sample plot at time *t* from the centroid obtained using all sampling times up to and including time *t*−1 from that lagoon.

The former of these measures focuses on detecting changes of a more gradual “press” nature over longer periods of time, the latter of these measures allows the baseline to move with (or be updated by) more recent observations and therefore focuses on detecting “pulse” changes (Anderson and Thompson 2004).

Non-parametric bootstrapping procedures were used to obtain statistical control limits: values obtained through time were sampled with replacement within each plot and deviations recalculated for each bootstrap sample. The 50th and 95th percentile of the distribution of deviations across all sites were obtained for each bootstrap sample. The mean of the bootstrap distribution of percentiles is used for plotting the statistical control limits (see Anderson and Thompson 2004 and Anderson 2008). Whereas the bootstrap distribution for a given percentile is not different for different time-points for the baseline procedure (1 above), the percentiles are time-point specific for the *t*−1 procedure (2 above).

## Results

**Figure 3:**
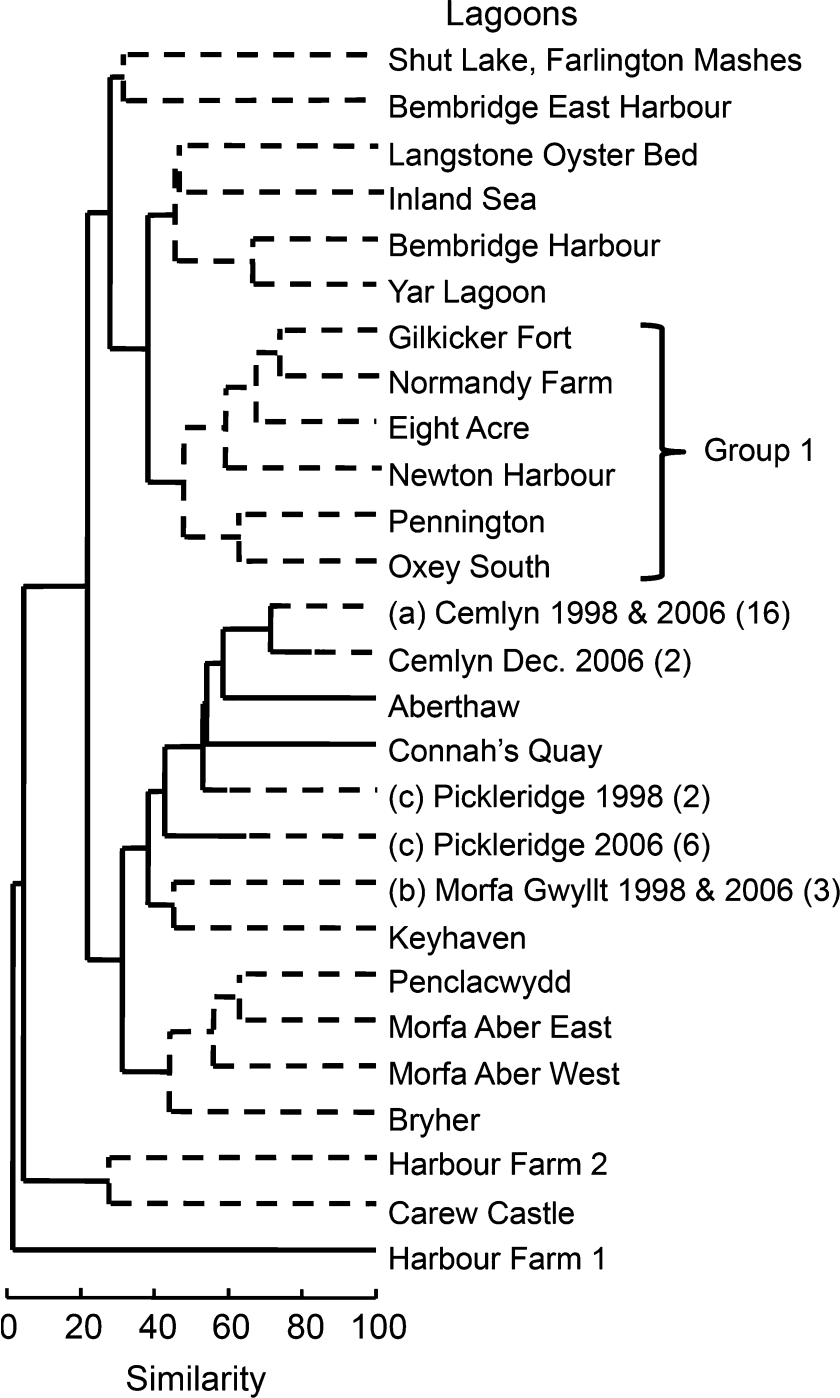
Relationship between the infauna of 25 lagoons and lagoon-like habitats based on Bray–Curtis similarities of fourth-root transformed community abundance data. Dotted lines indicate significant clusters (*P* < 0.05) arising from a Similarity Profile permutation routine. Clusters formed by multiple samples exclusively from one lagoon have been collapsed to aid interpretation (number of samples in parentheses). Group 1 is the largest group of similar lagoons from within 30 km of each other in the Solent (see blow-up in Fig. 1). Letter codes refer to protected lagoons studied over time (a–c).

Multivariate analysis of the 25 lagoons and lagoon-like habitats showed some structure (SIMPROF, *P* < 0.05; Fig. 3), that is some communities in different sites were similar. Some, however, including the three lagoons in the time series study, showed very little similarity with any of the other sites, even when resampled in different years or seasons (Fig. 3: clusters a–c). The exception was Morfa Gwyllt which, on two occasions (1998 and 2006), showed similarities with the sample from Keyhaven lagoon. Sources of taxonomic and sampling bias between sites had been systematically eliminated prior to analysis (see Data treatment and statistical analysis) and the taxa that characterized Cemlyn, Pickleridge, and Morfa Gwyllt clusters (Fig. 3) were not problematic to identify or to collect (see Tables S1 & S2 in Supporting Information). The mostly exclusive clustering of these three lagoons was therefore considered genuine. Comparing the largest group of lagoons (Fig. 3; Group 1) to Cemlyn, for example, showed that they shared about half of the same species at differing abundances and, of the remaining taxa, half were lagoonal specialists (see Table S2).

Between 2006 and 2010, 30 taxa were recorded at Cemlyn, 40 at Pickleridge, and 14 at Morfa Gwyllt. At all three protected lagoons there were highly significant temporal effects in the community composition (pseudo *F* = 3.14, *P* = 0.0002; *F* = 2.27, *P* = 0.009; *F* = 3.16, *P* = 0.0002 for Cemlyn, Pickleridge, and Morfa Gwyllt, respectively). There was also a highly significant spatial effect due to the sample plots at Cemlyn and Pickleridge (pseudo *F* = 19.80, *P* = 0.0002; *F* = 7.52, *P* = 0.0001 for Cemlyn and Pickleridge, respectively). Temporal changes were not consistent throughout Cemlyn and Pickleridge lagoons as indicated by significant interaction terms (pseudo *F* = 3.34, *P* = 0.0001; *F* = 4.39, *P* = 0.0001; *F* = 4.39, *P* = 0.0001 for Cemlyn and Pickleridge, respectively). For all the lagoons there was therefore compelling evidence of substantial temporal and spatial variation in the infaunal communities, but there were no observations or reports of anthropogenic impacts throughout the 5-year study despite these being sites frequented by the authors and seasonal wardens. Estimates of each of the components of variation in the model were derived from expected mean squares in the PERMANOVA output and can be used to compare the relative importance of the terms in the model (see Anderson et al. 2008). In any of the lagoons, temporal or spatial factors in the model only explained 10–15% of the total variance (Table S3). The PERMDISP test for homogeneity of multivariate dispersion showed that significant variability identified in PERMANOVA analyses over time were attributable to species and abundances values and not differences in dispersion of these data (not significant: *F* = 1.18, *P*(perm.) = 0.44; *F* = 2.51, *P* = 0.10; *F* = 3.23, *P* = 0.11 for Cemlyn, Pickleridge, and Morfa Gwyllt, respectively). Pair-wise comparisons between years in each protected area lagoon showed that more than 70% of the possible comparisons varied significantly (Table 1).

**Table 1:** Test statistics and significance values of pair-wise comparisons (PERMANOVA) between years in three protected lagoon sites (based on 9999 permutations)

		2006	2007	2008	2009
*t*					
2007	C	1.59	–	–	–
	P	1.82	–	–	–
	MG	2.14	–	–	–
2008	C	1.71	1.44	–	–
	P	2.14	1.80	–	–
	MG	1.68	0.63	–	–
2009	C	2.15	1.90	1.75	–
	P	n/a	n/a	n/a	–
	MG	1.83	0.81	0.56	–
2010	C	1.72	1.28	1.78	2.44
	P	1.19	1.52	1.12	n/a
	MG	2.59	1.81	1.69	1.72
*P*(perm.)					
2007	C	0.02*	–	–	–
	P	0.05*	–	–	–
	MG	0.01*	–	–	–
2008	C	0.02*	0.22	–	–
	P	0.03*	0.03*	–	–
	MG	0.04*	0.80	–	–
2009	C	0.02*	0.02*	0.05*	–
	P	n/a	n/a	n/a	–
	MG	0.01*	0.64	0.80	–
2010	C	0.02*	0.07	0.02*	0.02*
	P	0.25	0.17	0.45	n/a
	MG	0.00*	0.03*	0.05	0.04*

C, Cemlyn; P, Pickleridge; MG, Morfa Gwyllt.

* Significant pair-wise comparisons at *P* < 0.05.

**Figure 4:**
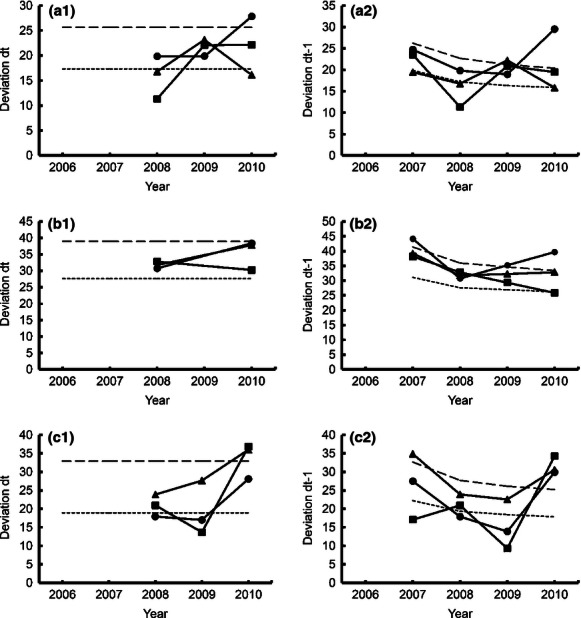
Multivariate control charts of 5 years of macrobenthic infauna data at sample plots 1, 2, and 3 (circle, square, triangle, respectively) in Cemlyn (a), Pickleridge (b), and Morfa Gwyllt (c) lagoons. Based on fourth-root transformed Bray–Curtis similarities of species abundance data using ‘distance to baseline’ from first 2 years data (left hand pane) and ‘distance to centroid’ using all previous years data (right hand pane). Control limits derived from the centroid of the time-series data, based on estimated means of 95th (long dash) and 50th (dash) bootstrapped percentiles (from 10000 bootstrap samples).

The 50% control limits (Fig. 4) indicate expected mean trajectories of the data across all the sites. For both control chart methods (press and pulse sensitive), plots 1 at Cemlyn and Pickleridge lagoons fall outside of the 95% control limit in 2010. Similarly, two of the pseudo-sample-plots at Morfa Gwyllt lagoon fall outside the control limits in the two charts (Fig. 4: c1, c2 for the same year). Sample plots that appear out of control in 2007 *t*−1 analyses were considered more likely artifacts because the graph will typically need time to stabilize (see Anderson and Thompson 2004).

## Discussion

The significant spatial and high levels of temporal variation in lagoon communities seen here are interpreted as inherent variability within these systems and the first null hypothesis, that there was no significant inherent spatial and temporal variability in the infaunal community, was therefore rejected. High-frequency sampling studies (e.g., Lucas et al. 2006) and long-term investigations in shallow, low volume, lagoons elsewhere (e.g., Munari et al. 2005; Coelho et al. 2007) have shown high physical variability and support the view that lagoons are potentially high-stress habitats suitable for euryhaline lagoonal specialist species (Bamber et al. 1992). In this study, the temporal and spatial changes in lagoon community probably reflect this inherent variability and the stochasticity of the salinity and temperature regimes in ways that we have yet to characterize (low components of variation in Table S3), but causality is beyond the scope of this article.

Some of the protected lagoon communities studied were also rare or unique: with little or no similarity with sites elsewhere (Figure 3). Pickleridge is probably more consistent relative to other sites than our data suggest (1998 and 2006 in Fig. 3) because it morphologically restabilized following the Sea Empress oil spill (February 2006) when the weir sill was reworked to prevent pollution (Moore 2006). Group 1 lagoon similarities (Fig. 3; Table S2) might be explained by close proximity (within 30 km of one another; Fig. 1) and corresponding biogeographic or coastal geo-morphology similarities that have led to common faunas, but differences in lagoonal specialists compared to the more geographically isolated and unique Cemlyn, Pickleridge, and Morfa Gwyllt.

The intensively studied coastal saline lagoons reported here are all within protected areas. Many published studies on the performance or condition of protected areas compare variables either 1) inside and outside the protected area and/or 2) over time scales, such as before and after reserve implementation or at various intervals thereafter (e.g., Gaston et al. 2008; Lester et al. 2009). This approach allows for well-formulated, tractable questions to be addressed (Nichols and Williams 2006; Lindenmayer et al. 2007; Lester et al. 2009; Lindenmayer and Likens 2010) with many studies conforming to BACI-like designs (e.g., Underwood 1997). Throughout the world, richness, abundance, and biomass of species have been compared inside and outside protected areas and over time using this approach (e.g., Caro 2002, 2003; Fabricius et al. 2003; Guidetti 2006, 2007; McClanahan et al. 2006; Rannestad et al. 2006; Setsaas et al. 2007; Harborne et al. 2008; Lester et al. 2009; Aburto-Oropeza et al. 2011). However, in this study, the rarity of comparable protected habitat precludes inside–outside studies or even comparison with other protected areas. Managers are nevertheless compelled to deal with ‘question-driven’ monitoring (cf. Lindenmayer and Likens 2010), perhaps about potential impacts, as well as to reassure themselves and others that the protected area is achieving its objectives (e.g., legislated reporting in Council Directive 92/43EEC, Article 17). Monitoring is also, however, experimentally difficult or inconclusive when confronted by statistically highly significant inherent changes in a system (as here). Arguably, the published science on protected areas consequently does not deal with many cases of rare and/or variable systems for these reasons and these factors may contribute to the poor understanding of the ecological performance of protected areas that Gaston et al. (2008) observed.

This study has, so far, been conducted over a relatively short period of time albeit detailed for this type of habitat and is nevertheless typical of resource-constrained monitoring programs. Multivariate control chart (MCC) analysis of the time-series data provides context, showing that while sample plots changed significantly in a conventional sense (cf. PERMANOVA results here), they were largely ‘in-control’ compared to statistically derived control limits from this study. The exception was 2010, when more than half of the sample plots from the three protected lagoons were ‘out of control’: this was probably genuine because it was the coldest December in 100 years (5°C below long-term average; Eden 2011) and the only occasion when ice was found during sampling. Overall therefore, the second null hypothesis is rejected because the MCC approach leads to different conclusions about significant variability with the possible exception of 2010. Temporal differences were generally not ecologically significant, but were instead a reflection of inherent variation in these systems. Anderson and Thompson (2004) proposed the use of the bootstrap estimates of empirical percentiles as a means of determining reasonable control limits for management and decision making and, in our problematic monitoring example, seem to provide a solution that is not distracted by what conventionally would be regarded as significant changes. Importantly, the MCC solution also does not require an inside-outside comparison that is an obstacle for the study of rare or unique protected habitats.

Elsewhere, univariate control charts have been used in studies monitoring contaminants, biomarkers, fish numbers, fish growth, and reptile abundance (Rounsefell and Bond 1950; Allen et al. 1997; Poulain 2001; Atkinson et al. 2003; Chèvre et al. 2003; Chapman and DeBruyn 2007; Trexler and Goss 2009). This study, however, is one of the few that has applied control charts to multivariate community data and/or protected areas (see also Anderson and Thompson 2004; Petitgas and Poulard 2009). Control charts provide “a useful objective and quantitative tool for identifying unusual events in ecological data sets” (Allen et al. 1997) and rapid visualization for stakeholders, managers, and the wider scientific community (Anderson and Thompson 2004; Chapman and DeBruyn 2007) especially those with little time, without a strong statistical background, but with a need to get an accurate understanding of the issue (Morrison 2008). Unlike traditional significance testing, one does not have to conclude that no difference exists over time, but rather that the observations are either within the specified range of acceptable values or not (Morrison 2008). Unlike regression analyses or BACI designs, MCC can also provide an early warning signal of a system that may be going “out-of-control” after a single time of sampling post impact (Anderson and Thompson 2004). Conversely, however, MCCs cannot deal with causality and are therefore ‘alarm signals’ that require further investigation (Chapman and DeBruyn 2007).

Assessment tools based on indices of infaunal communities are routinely used in European waters (Council of the European Communities 1992; European Commission 2000), which has spawned numerous reviews of their relative merits (e.g., Ruellet and Dauvin 2007; Puente and Diaz 2008; Pinto et al. 2009; Kröncke and Reiss 2010). There are two problems with using these kinds of index-based analyses in the assessment of low volume lagoons. The first is the difficulty of defining a realistic set of habitat type-specific reference conditions that finds a sensible balance between having a large array of types with low internal variance or a few types that have high intratype variation (Basset et al. 2006). The second problem is that the majority of indices of anthropogenic stress in coastal ecosystems are based on a model where high abundances of stress-tolerant species and low diversity and evenness occur when conditions are unfavorable: this presumption distorts assessments of the ecological status of lagoons, which are naturally low diversity, high abundance, stress-tolerant communities (Breber et al. 2007; Magni et al. 2008). It might take years or even decades of data to understand the variability in both habitat conditions and community structure in lagoons (Allen et al. 1997) before generic acceptable and unacceptable conditions could be described. In contrast, MCC are an easy to use tool that allows the analysis of rare, variable habitats in a way that can inform protected area management decisions and can be fit for purpose in a relatively short time frame. We therefore contend that MCCs have wide application in protected area and environmental management.

## Supporting Information

**Table S1.** Species characterizing clusters of samples from Cemlyn, Pickleridge, and Morfa Gwyllt lagoons

**Table S2.** Species differentiating clusters of samples between: (A) Pickleridge in 1998 and 2006, (B) Cemlyn lagoon and Group 1 lagoons

**Table S3.** Components of variation in the communities at Cemlyn, Pickleridge, and Morfa Gwyllt
