# Supplementary material for: A tool for protected area management: multivariate control charts ‘cope’ with rare variable communities
Source: Ecol Evol. 2013 May 1;3(6):1667–76. doi: 10.1002/ece3.585 (PMC3686200; doi:10.1002/ece3.585)
Supplement: Supplementary file 1 [file ece30003-1667-SD1.docx]

**Table S1.** Species characterising clusters of samples from Cemlyn, Pickleridge and Morfa Gwyllt lagoons (Fig. 3: SIMPROF analysis) based on SIMPER analysis of fourth-root-transformed abundance data

| 1. **Cemlyn (1998 & 2006)**   Average similarity: 77.84  **Species** | **Average Abundance** | **Average Similarity** | **Sim/SD** | **Contribution (%)** | **Cumulative (%)** |
| --- | --- | --- | --- | --- | --- |
| ***Ventrosia ventrosa*** | 4.64 | 15.50 | 7.50 | 19.92 | 19.92 |
| ***Heterochaeta costata*** | 3.50 | 12.01 | 9.92 | 15.43 | 35.35 |
| *Corophium volutator* | 2.82 | 9.27 | 5.55 | 11.91 | 47.25 |
| *Hydrobia ulvae* | 2.69 | 9.20 | 9.52 | 11.82 | 59.07 |
| Enchytraeidae | 1.97 | 5.78 | 2.33 | 7.42 | 66.49 |
| *Polydora cornuta* | 1.89 | 5.77 | 5.02 | 7.41 | 73.91 |
| *Hediste diversicolor* | 1.76 | 5.68 | 6.32 | 7.30 | 81.21 |
| *Manayunkia aestuarina* | 1.33 | 3.71 | 1.71 | 4.77 | 85.97 |
| *Pygospio elegans* | 1.29 | 2.92 | 1.25 | 3.75 | 89.72 |
| *Tubificoides* spp. | 1.14 | 2.37 | 0.90 | 3.05 | 92.77 |
|  |  |  |  |  |  |
| 1. **Morfa Gwyllt (1998 & 2006) + Keyhaven**   Average similarity: 55.92  **Species** |  |  |  |  |  |
| *Corophium volutator* | 4.13 | 16.14 | 9.87 | 28.85 | 28.85 |
| ***Heterochaeta costata*** | 3.99 | 13.80 | 8.19 | 24.68 | 53.54 |
| *Hediste diversicolor* | 3.28 | 9.26 | 1.81 | 16.55 | 70.09 |
| Enchytraeidae | 2.63 | 6.04 | 0.90 | 10.80 | 80.89 |
| ***Lekanesphaera hookeri*** | 1.67 | 6.03 | 2.36 | 10.78 | 91.67 |
|  |  |  |  |  |  |
| 1. **Pickleridge (2006)**   Average similarity: 66.96  **Species** |  |  |  |  |  |
| ***Corophium insidiosum*** | 3.45 | 13.46 | 7.82 | 20.10 | 20.10 |
| *Hediste diversicolor* | 2.55 | 10.12 | 11.84 | 15.11 | 35.21 |
| *Polydora cornuta* | 2.36 | 8.87 | 22.86 | 13.25 | 48.46 |
| *Hydrobia ulvae* | 1.65 | 6.21 | 12.77 | 9.27 | 57.73 |
| *Tharyx* sp. A | 1.69 | 4.89 | 4.96 | 7.31 | 65.04 |
| *Streblospio shrubsolii* | 1.59 | 4.59 | 1.34 | 6.86 | 71.90 |
| *Tubificoides* spp. | 1.66 | 3.47 | 0.77 | 5.19 | 77.09 |
| *Melita palmate* | 1.21 | 3.04 | 1.21 | 4.55 | 81.63 |
| ***Cerastoderma glaucum*** | 0.97 | 3.01 | 1.33 | 4.49 | 86.13 |
| ***Heterochaeta costata*** | 1.39 | 2.51 | 0.74 | 3.75 | 89.87 |
| Enchytraeidae | 1.23 | 2.40 | 0.75 | 3.58 | 93.45 |

*Species considered ‘lagoonal specialists’ in the UK are in bold text (e.g. Bamber *et al*. 1992)

†Taxonomy follows World Register of Marine Species where authorities can be found: <http://www.marinespecies.org/about.php>
