# Supplementary material for: A tool for protected area management: multivariate control charts ‘cope’ with rare variable communities
Source: Ecol Evol. 2013 May 1;3(6):1667–76. doi: 10.1002/ece3.585 (PMC3686200; doi:10.1002/ece3.585)
Supplement: Supplementary file 2 [file ece30003-1667-SD2.docx]

**Table S2.** Species differentiating clusters of samples between: a. Pickleridge in 1998 and 2006, b. Cemlyn lagoon and Group 1 lagoons. Based on SIMPROF clusters (in Fig. 3) using SIMPER analysis on fourth root abundance data.

| **(A) Pickleridge lagoon** | **2006** | **1998** |  |  |  |  |
| --- | --- | --- | --- | --- | --- | --- |
| Average similarity: 53.26  **Species** | **Average Abundance** | **Average Abundance** | **Average Dissimilarity** | **Diss/SD** | **Contribution (%)** | **Cumulative (%)** |
| ***Corophium insidiosum*** | 3.45 | 0.00 | 7.71 | 6.68 | 14.47 | 14.47 |
| *Mya arenaria* | 0.58 | 2.80 | 4.91 | 2.90 | 9.21 | 23.68 |
| ***Heterochaeta costata*** | 1.39 | 3.04 | 3.96 | 1.54 | 7.44 | 31.12 |
| Enchytraeidae | 1.23 | 1.76 | 3.84 | 1.51 | 7.21 | 38.33 |
| *Tubificoides* spp. | 1.66 | 0.00 | 3.70 | 1.27 | 6.96 | 45.28 |
| *Tharyx* sp. A | 1.69 | 0.00 | 3.70 | 2.13 | 6.94 | 52.22 |
| *Pygospio elegans* | 0.17 | 1.53 | 3.19 | 1.04 | 5.98 | 58.20 |
| ***Ventrosia ventrosa*** | 0.00 | 1.27 | 3.04 | 0.95 | 5.71 | 63.91 |
| *Melita palmata* | 1.21 | 0.00 | 2.65 | 1.60 | 4.98 | 68.89 |
| *Streblospio shrubsolii* | 1.59 | 1.09 | 2.40 | 1.18 | 4.51 | 73.40 |
| *Abra tenuis* | 0.91 | 0.00 | 1.98 | 1.27 | 3.71 | 77.11 |
| *Cyathura carinata* | 0.77 | 0.00 | 1.77 | 0.91 | 3.32 | 80.42 |
| *Polydora cornuta* | 2.36 | 2.34 | 1.46 | 1.89 | 2.74 | 83.16 |
| *Hydrobia ulvae* | 1.65 | 2.21 | 1.39 | 1.79 | 2.61 | 85.76 |
| ***Cerastoderma glaucum*** | 0.97 | 1.60 | 1.38 | 1.35 | 2.58 | 88.35 |
| *Corophium volutator* | 0.00 | 0.59 | 1.23 | 0.95 | 2.31 | 90.66 |

| **(B) Cemlyn lagoon & Group 1 lagoons** | **Cem.** | **Grp1** |  |  |  |  |
| --- | --- | --- | --- | --- | --- | --- |
| Average dissimilarity = 77.28  **Species** | **Average Abundance** | **Average Abundance** | **Average Dissimilarity** | **Diss/SD** | **Contribution (%)** | **Cumulative (%)** |
| ***Ventrosia ventrosa*** | 4.64 | 0.78 | 8.27 | 3.34 | 10.70 | 10.70 |
| ***Heterochaeta costata*** | 3.50 | 0.00 | 7.54 | 5.92 | 9.76 | 20.46 |
| *Hydrobia ulvae* | 2.69 | 0.00 | 5.79 | 6.31 | 7.49 | 27.95 |
| *Corophium volutator* | 2.82 | 0.93 | 4.34 | 1.74 | 5.61 | 33.56 |
| Enchytraeidae | 1.97 | 0.00 | 4.21 | 2.81 | 5.45 | 39.01 |
| ***Nematostella vectensis*** | 0.00 | 1.91 | 4.03 | 1.84 | 5.22 | 44.23 |
| ***Corophium insidiosum*** | 0.95 | 2.34 | 3.61 | 1.77 | 4.67 | 48.90 |
| ***Idotea chelipes*** | 0.22 | 1.81 | 3.47 | 2.47 | 4.49 | 53.40 |
| ***Gammarus insensibilis*** | 0.00 | 1.62 | 3.43 | 3.96 | 4.43 | 57.83 |
| *Polydora cornuta* | 1.89 | 1.17 | 2.92 | 1.57 | 3.78 | 61.61 |
| *Manayunkia aestuarina* | 1.33 | 0.00 | 2.86 | 2.20 | 3.70 | 65.32 |
| *Microdeutopus gryllotalpa* | 0.00 | 1.29 | 2.85 | 1.19 | 3.68 | 69.00 |
| *Hediste diversicolor* | 1.76 | 0.68 | 2.84 | 1.80 | 3.68 | 72.68 |
| *Pygospio elegans* | 1.29 | 0.00 | 2.75 | 1.54 | 3.55 | 76.23 |
| *Capitella capitata* | 0.19 | 1.30 | 2.65 | 1.20 | 3.43 | 79.67 |
| *Tubificoides* spp. | 1.14 | 2.25 | 2.63 | 1.36 | 3.40 | 83.07 |
| ***Cerastoderma glaucum*** | 0.06 | 1.19 | 2.46 | 1.70 | 3.18 | 86.25 |
| ***Lekanesphaera rugicauda*** | 0.00 | 1.06 | 2.15 | 1.39 | 2.79 | 89.03 |
| *Streblospio shrubsolii* | 0.90 | 0.25 | 1.92 | 1.07 | 2.49 | 91.53 |

*Species considered ‘lagoonal specialists’ in the UK (e.g. Bamber *et al*. 1992) are in bold text

†Taxonomy follows World Register of Marine Species where authorities can be found: <http://www.marinespecies.org/about.php>
