# Supplementary material for: A tool for protected area management: multivariate control charts ‘cope’ with rare variable communities
Source: Ecol Evol. 2013 May 1;3(6):1667–76. doi: 10.1002/ece3.585 (PMC3686200; doi:10.1002/ece3.585)
Supplement: Supplementary file 3 [file ece30003-1667-SD3.docx]

**Table S3.** Estimates of the components of variation in the infaunal communities 2006 - 2010 at Cemlyn, Pickleridge and Morfa Gwyllt lagoonal sites

| **a. Cemlyn lagoon** |  |  |
| --- | --- | --- |
| **Source** | **Estimate** | **Sq. root** |
| Variation (Year) | 105.78 | 10.29 |
| Variation (Sample plot) | 167.04 | 12.93 |
| Variation (Sample plot/Year) | 103.79 | 10.19 |
| Variation (Residual) | 177.71 | 13.33 |
| **b. Pickleridge lagoon** |  |  |
| **Source** | **Estimate** | **Sq. root** |
| Variation (Year) | 210.30 | 14.50 |
| Variation (Sample plot) | 184.50 | 13.58 |
| Variation (Sample plot/Year) | 384.05 | 19.60 |
| Variation (Residual) | 565.97 | 23.79 |
| **c. Morfa Gwyllt lagoon** |  |  |
| **Source** | **Estimate** | **Sq. root** |
| Variation (Year) | 191.10 | 13.82 |
| Variation (Residual) | 883.50 | 29.72 |
